# Comparative Analysis of the Cytology and Transcriptomes of the Cytoplasmic Male Sterility Line H276A and Its Maintainer Line H276B of Cotton (*Gossypium barbadense* L.)

**DOI:** 10.3390/ijms18112240

**Published:** 2017-10-25

**Authors:** Xiangjun Kong, Dongmei Liu, Xiaofang Liao, Jie Zheng, Yong Diao, Yiding Liu, Ruiyang Zhou

**Affiliations:** 1 Key Laboratory of Plant Genetics and Breeding, College of Agriculture, Guangxi University, Nanning 530006, China; phdkongxiangjun@gmail.com (X.K.); phdliaoxiaohua@gmail.com (X.L.); summerjackk@gmail.com (J.Z.); dioryongdr@gmail.com (Y.D.); phdliuyiding@gmail.com (Y.L.); 2 Key Laboratory of Plant-Microbe Interactions, Department of Life Science and Food, Shangqiu Normal University, Shangqiu 476000, China; liudongmei1997@gmail.com

**Keywords:** cotton (*Gossypium barbadense* L.), cytoplasmic male sterility (CMS), microspore development, transcriptome, differentially expressed genes (DEGs)

## Abstract

In this study, the tetrad stage of microspore development in a new cotton (*Gossypium barbadense* L.) cytoplasmic male sterility (CMS) line, H276A, was identified using paraffin sections at the abortion stage. To explore the molecular mechanism underlying CMS in cotton, a comparative transcriptome analysis between the CMS line H276A and its maintainer line H276B at the tetrad stage was conducted using an Illumina HiSeq 4000 platform. The comparison of H276A with H276B revealed a total of 64,675 genes, which consisted of 59,255 known and 5420 novel genes. An analysis of the two libraries with a given threshold yielded a total of 3603 differentially expressed genes (DEGs), which included 1363 up- and 2240 down-regulated genes. Gene Ontology (GO) annotation showed that 2171 DEGs were distributed into 38 categories, and a Kyoto Encyclopedia of Genes and Genomes (KEGG) analysis showed that 2683 DEGs were classified into 127 groups. Thirteen DEGs were randomly selected and detected by quantitative reverse-transcribed PCR (qRT-PCR), and the results indicated that the transcriptome sequencing results were reliable. The bioinformatic analysis results in conjunction with previously reported data revealed key DEGs that might be associated with the male sterility features of H276A. Our results provide a comprehensive foundation for understanding anther development and will accelerate the study of the molecular mechanisms of CMS in cotton.

## 1. Introduction

Cotton (*Gossypium barbadense* L.) is an important economic crop that is grown for its textile fibers and edible oil. However, cotton development is suppressed by low yield. The use of heterosis is a potential technical breakthrough with respect to increasing future yields. Cytoplasmic male sterility (CMS) constitutes the basis of heterosis and has been widely used in rice, maize, and sorghum [1]. However, the use of heterosis in cotton is scarce due to the limited existence of CMS cotton germplasms. Studies on the molecular mechanisms of CMS could provide an important theoretical basis for the generation of CMS lines.

At the cytological level, CMS results from non-functional pollen in which microspore degradation occurs during various stages of microspore development. Microspore degradation is associated with abnormal programmed cell death (PCD) of the tapetum, which is responsible for supplying nutrition to the developing microspore [2]. Advanced or delayed degradation of the tapetum can lead to the degradation of microspores in plants, such as *Brassica napus* [3], cabbage [4], and *Capsicum annuum* [5]. Microspore development is a complex process, and identification of the abortion stage during exploration of the molecular mechanisms of CMS could facilitate elimination of the interference from unrelated genes.

Transcriptomics is used to investigate the transcription and expression at the RNA level of genomes at specific developmental stages or under specific physiological conditions [6]. Transcriptome sequencing (RNA-Seq) can provide information on the expression of thousands of genes in a single experiment and has greatly promoted the development of functional genomics. CMS is caused by mitochondrial genes coupled with nuclear genes [7] and is considered a complicated biological process that involves many metabolic pathways. Therefore, comparative transcriptome analyses have been extensively used to study the molecular mechanisms of CMS in plants, including the kenaf CMS line P3A [8], soybean CMS line NJCMS1A [9], Welsh onion CMS line 64-2 [10], and cabbage CMS line PM [4].

*Gossypium barbadense* H276A is a novel cotton CMS line. In the present study, H276A was generated by transferring the kenaf *HcPDIL5-2a* gene to wild-type H276B using the pollen tube pathway method. Because the CMS line H276A is a mutant of its maintainer line H276B, they are iso-genic at the mitochondrial genome level. In studies of the mechanism of cotton CMS, the non CMS-related redundant genetic information caused by evolution of the mitochondrial genome can be excluded; however, this information is valuable for the study of molecular mechanisms of CMS in cotton. The cytological characteristics and the molecular mechanism of the CMS line H276A are unknown. Therefore, this study comparatively analyzed the cytological characterization of the CMS line H276A and its maintainer line H276B to better understand the mechanism of abortion in H276A. Furthermore, transcriptome profiles of the floral buds of both materials at the abortion stage were generated based on the cytological results. Our data will accelerate elucidation of the molecular mechanism of the CMS line H276A of cotton.

## 2. Results

### 2.1. Determination of the Pollen Abortion Stage in CMS Line H276A

**Figure 1:**
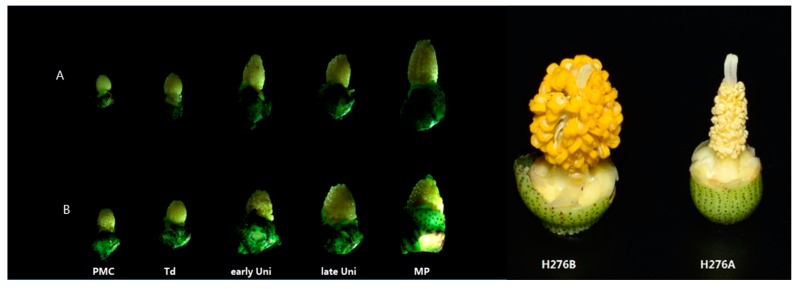
Phenotype of the floral buds of the cytoplasmic male sterility (CMS) line H276A and its maintainer H276B: (**A**): CMS line H276A; (**B**): Maintainer line H276B. Notes: PMC, pollen mother cell stage; Td, tetrad stage; early Uni, early uninucleate; late Uni, late uninucleate; MP, mature pollen stage.

Floral organs play an important role in the plant reproductive process. Abnormal stamens, in which there is no pollen or anther indehiscence occurs, always lead to CMS. The phenotypes of the floral organs of the CMS line H276A and its maintainer line H276B were found to be similar during the microspore developmental process. However, during the mature period, the filaments of H276A were shorter than those of H276B, and the anthers of the former were more wrinkled. (Figure 1).

**Figure 2:**
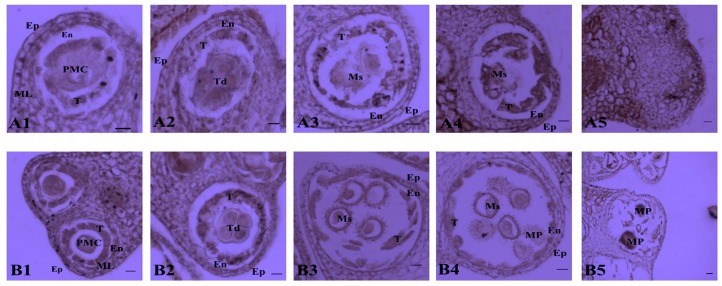
Comparative analysis of anther development between H276A (**A1**–**A5**) and H276B (**B1**–**B5**). Notes: Bar = 50 μm for all of the stage. PMC, pollen mother cell; Ep, epidermis; En, endothecium; ML, middle layer; T, tapetum; Ms, microspore; MP, mature pollen.

**Figure 3:**
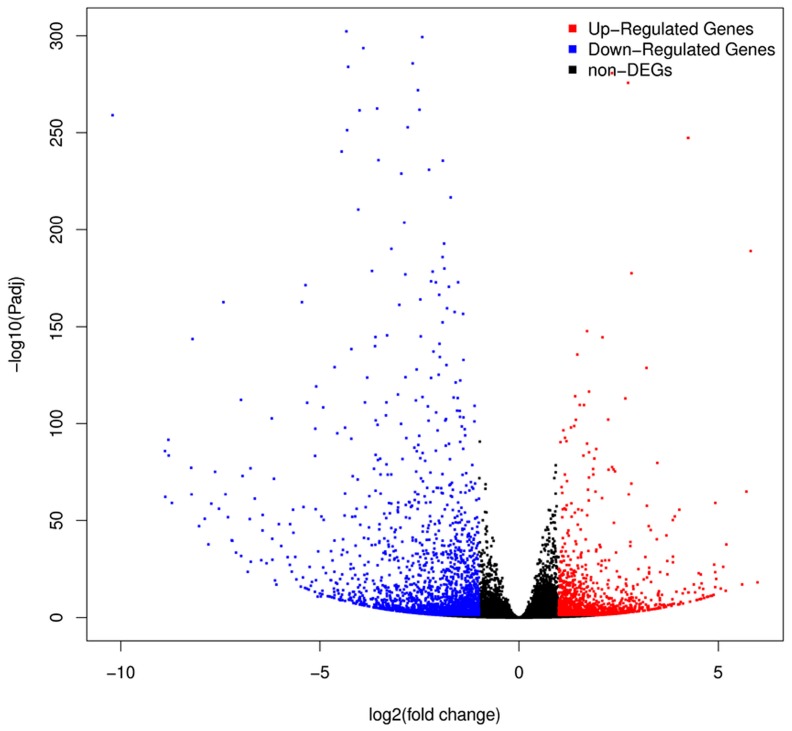
Comparison of gene expression levels between H276A and H276B according to the DEseq2 method.

Microspore observations of different-sized floral buds were used to detect the pollen abortion stage of the CMS line H276A. At the pollen mother cell (PMC) stage (3–4 mm), no differences between the sterile and fertile lines were observed (Figure 2A1,B1), and the pollen sacs consisted of an epidermis, endothecium, middle layer, tapetum and PMC. At the tetrad (4–5 mm) stage (Figure 2A2,B2), the PMC formed tetrads after meiosis, and these were surrounded by the tapetum in the fertile line. However, the tetrads in the sterile line were vacuolar, and the nuclei of the tetrads gradually dissolved. At the early uninucleate stage (5–6 mm), the nucleus in the normal fertile line H276B was squeezed to the edge of the cell by the central vacuole, and the tapetum began to dissolve (Figure 2B3). However, in the sterile line H276A, the tapetum became integrated, and the microspores were degraded (Figure 2A and Figure 3). At the late uninucleate stage (6–7 mm), the microspores in the fertile line differentiated into pollen grains (Figure 2B4), whereas the microspores of H276A disappeared, and the tapetum was calmly integrated (Figure 2A4). At the mature pollen grain stage (>7 mm), all microspores in H276B had become mature pollen (Figure 2B5). In contrast, the pollen sacs of H276A were wrinkled, and the microspores disappeared (Figure 2A5). The results of these microspore observations indicated that degradation of the nuclei of the microspores in the tetrad stage and degradation of the tapetal cells did not occur during microspore development in H276A, leading to CMS. Specifically, we inferred that the tetrad cells in H276A did not acquire sufficient nutrition during microspore development, which caused microspore degradation and CMS. The analysis of the observations of microspore development suggested that the tetrad stage was the abortion stage in the sterile line. Therefore, floral buds from the tetrad stage were collected for further study of the molecular mechanism of CMS.

### 2.2. Transcriptome Sequencing and Genome Mapping

In the present study, a transcriptome sequencing analysis of tetrad-stage floral buds of the cotton CMS line H276A and its maintainer line H276B was conducted using an Illumina HiSeq 4000 sequencer. Each sequencing read was 150 bp in length. The Q20 percentages exceeded 98% in each sample. Total raw reads consisting of 155.66 Mb and 155.46 were obtained for H276A and H276B, respectively, and total clean reads comprising 129.59 Mb and 133.2 Mb were generated after the raw reads were filtered, respectively. All clean reads were mapped to the cotton reference genome, and on average, 75.09% of the reads were mapped in each library. The uniformity of the mapping results for each sample suggested that the samples are comparable. After genome mapping, we conducted novel transcript prediction and identified 36,680 novel transcripts, including 30,147 coding transcripts and 6533 noncoding transcripts. The numbers of novel transcripts from each sample are presented in Table 1.

### 2.3. Analysis of DEGs between H276A and H276B

After performing the novel transcript predictions, we merged the novel coding transcripts and the cotton reference genome to obtain a complete reference. All clean reads were then matched to the complete reference genome with Bowtie 2 [11]. The results showed that an average of 57,154 genes (85,544 transcripts) were detected from each sample (Table 1). The gene expression level of each sample was calculated and is shown in Supplement 2.

DEGs between samples were identified using the PoissonDis method with the following threshold values: FDR ≤ 0.001 and |log_2_ (fold change, FC)| ≥ 1. On average, 3537 DEGs from each comparative group were identified, and three biological replicates of both materials were merged using the DEseq2 method. A total of 64,675 genes (Supplement 3), comprising 59,255 known and 5420 novel genes, were obtained from the H276A vs. H276B comparison. Furthermore, 3603 DEGs were identified from both merged libraries using the following threshold values: *p* ≤ 0.05 and |log_2_ (FC)| ≥ 1. These DEGs included 1563 up-regulated and 2240 down-regulated genes in H276A compared with H276B, respectively (Figure 3 and Supplement 4). The subsequent bioinformatics analysis was based on these DEGs.

**Figure 4:**
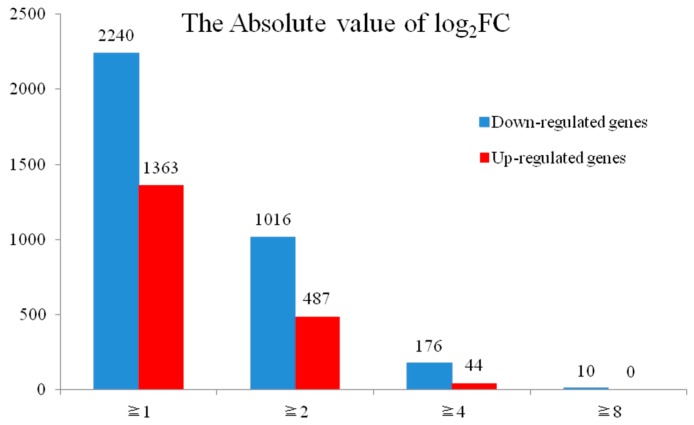
Distribution of the differentially expressed genes (DEGs) between H276A and H276B identified using different fold-change thresholds.

To more accurately assess the genetic differences, the DEGs between the H276A and H276B libraries were classified by fold-change values (Figure 4). As shown in Figure 4, 10 down-regulated genes were identified when the threshold was set to an eightfold difference. These highly down-regulated genes encoded lipid transfer proteins, aquaporin NIP7-1, endopolygalacturonase, ATPase subunit 8, plasma membrane-type-like proteins, and glucan endo-1,3-β-glucosidase, which might play vital roles during the pollen abortion process (Supplement 5).

### 2.4. GO Annotation and Pathway Analysis of DEGs

**Figure 5:**
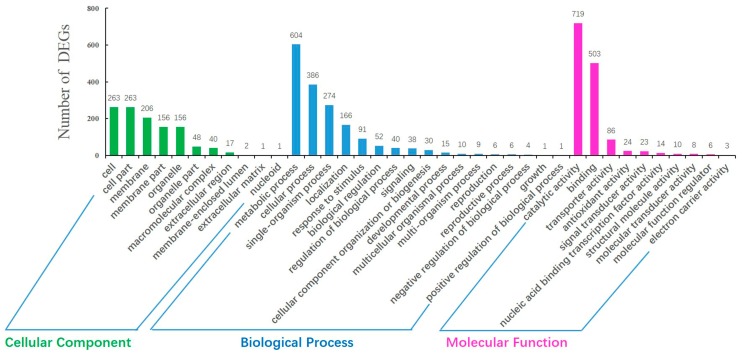
Gene ontology classification of DEGs in the H276A vs. H276B library.

We performed GO classification and functional enrichment of 3603 DEGs. As shown in Figure 5, 2171 DEGs were distributed among 38 GO items. Regarding cellular components, cell and cell part were associated with the highest number of DEGs, followed by membrane, membrane part, and organelle. The analysis of biological processes revealed that the highest number of DEGs were associated with metabolic process, followed by cellular process, single-organism process and localization response to stimulus. In terms of molecular function, most DEGs were associated with catalytic activity and binding transporter activity.

In contrast, the enrichment of GO terms in the DEGs was explored, with corrected *p* ≤ 0.05 indicating significance. For the cellular component category, significantly enriched GO terms included membrane (206 of 435, 47.7%), chloroplast (28 of 435, 6.4%), intrinsic component of membrane (139 of 435, 32.0%), membrane part (156 of 435, 35.9%), chloroplast envelope (7 of 435, 1.6%), and plastid (54 of 435, 12.4%). Analysis of the biological process category identified three significantly enriched GO terms, namely, peptide transport (13 of 802, 1.6%), amide transport (13 of 802, 1.6%), and nitrogen compound transport (21 of 802, 2.1%). The molecular function category analysis showed that 16 GO terms were significantly enriched, and these included oxidoreductase activity (205 of 934, 21.9%) and catalytic activity (719 of 934, 7.0%). Detailed results of these analyses are presented in Supplement 6.

To identify the metabolic networks and biological pathways of the DEGs, we performed Kyoto Encyclopedia of Genes and Genomes (KEGG) pathway classification and functional enrichment of DEGs, and 2683 DEGs were annotated and classified into 127 groups. In addition, 29 KEGG pathways were significantly enriched, with *Q* ≤ 0.05 (Table 2), and the top five of these pathways were metabolic pathways (ko01100), biosynthesis of secondary metabolites (ko01110), amino sugar and nucleotide sugar metabolism (ko00520), pentose and glucuronate interconversions (ko00040), and galactose metabolism (ko00052). These pathways associated with the identified DEGs might provide a basis for further analyses of genes involved in CMS.

### 2.5. Confirmation of DEGs by qRT-PCR

To evaluate the reliability of the Illumina sequencing technology, 13 DEGs (six up-regulated and seven down-regulated genes) were randomly selected and detected by qRT-PCR. Our data showed that the expression patterns of the 13 DEGs were in accordance with the RNA-Seq data, indicating that the Illumina sequencing data were reliable (Table 3). However, the fold change of some DEGs, such as LOC107903993 and LOC107940452, differed, and this difference might be due to the different principles employed by the two techniques [12]: RNA-Seq measures absolute quantification, whereas qRT-PCR was used to measure relative expression in this study.

## 3. Discussion

Previous studies have investigated cotton CMS, and cotton CMS lines and maintainer lines from different subspecies, such as CMS-D2 [13] and CMS-D8 [14], have been used in comparative analyses. Significant differences have been identified, but these differences might be associated with evolutionary divergence and have no relationship with CMS. In the present study, the CMS line H276A and its maintainer line H276B are isogenic with respect to not only the nuclear genome but also the mitochondrial genome. Therefore, the CMS line H276A makes it convenient to study the molecular mechanism of CMS in cotton.

### 3.1. Cytological Characteristics of Pollen Abortion

The microspore abortive process differs from other previously identified sterility mechanisms. In cotton CMS-D2, microspore abortion occurs during various development periods of sporogenous cells and the PMC. Abortion characteristics result from abnormal chromosome behavior, the formation of multiple nuclear within a cell and high vacuolization of the cytoplasm [15]. However, in the present study, abnormal microspore development was detected at the tetrad stage. In the CMS line H276A, the microspore disappeared at the late uninucleate stage, and the tapetum was integrated during the process of microspore development. The results were consistent with those found for the *Brassica napus* CMS line SaNa-1A [3], suggesting that microspore abortion is caused by abnormal PCD of the tapetum. In addition, the microspore might not obtain adequate nutrition from the tapetum during development, leading to CMS.

### 3.2. DEGs Related to the Tricarboxylic Acid (TCA) Cycle and Oxidative Phosphorylation

Pollen development is an energy-consuming process, and decreased energy supply might cause pollen abortion [16]. Mitochondria are an important cellular site for numerous energy-related metabolic pathways, including the TCA cycle, respiratory electron transfer, and oxidative phosphorylation [17,18]. Plant CMS is associated with the altered expression of TCA-related genes, and most DEGs are down-regulated. For example, a cabbage CMS transcriptome study identified 12 DEGs involved in the TCA cycle, and eight of these were down-regulated [4]. In the present study, 12 DEGs (11 down-regulated genes) that participate in the TCA cycle were identified (Supplement 7). Among these DEGs, LOC107936128 (−1.95 down-regulated) and LOC107927299 (−1.35 down-regulated) encode different subunits of citrate synthase. Citrate synthase is a critical enzyme of the TCA cycle that catalyzes the reaction of acetyl-CoA and oxaloacetic acid to generate citrate [3].

The respiratory electron transfer chain and oxidative phosphorylation are major pathways in ATP synthesis. These pathways include key protein subunits encoded by the mitochondrial genome [19]. Recombination and rearrangement of the mitochondrial genome can cause the formation of chimeric genes that influence the expression of mitochondrial or nuclear genes involved in respiration/ATP synthesis, leading to CMS [7]. Cytochrome c oxidase, a key enzyme of the mitochondrial inner membrane, is related to plant CMS. In the beet G-CMS line, the altered expression of *cox2* decreases cytochrome c oxidase activity by 50%, which leads to CMS [20]. In a pepper CMS line, the chimeric gene *orf456* was found to be inserted into the 3′-end of the *cox2* gene and might alter the fertility characterization [21]. In addition, some CMS genes are closely related to ATPase genes, such as *orf125–atp8* in radish CMS-Kos [22]. In the present study, 34 DEGs were associated with the electron transport chain and oxidative phosphorylation (Supplement 8). Among these DEGs, cytochrome c oxidase subunit 3 and ATPase subunit 8 were found to be down-regulated by −3.44- and −8.72-fold, respectively, in the CMS line H276A. Therefore, the altered expression of *cox3* and *atp8* might be associated with cotton CMS.

### 3.3. DEGs Associated with Pentatricopeptide Repeat (PPR) Proteins

The family of PPR proteins in plants comprises approximately 450 members and are targeted to mitochondria or chloroplasts [8]. PPR proteins are involved in post-transcriptional processes, including RNA editing, splicing, cleavage, degradation, and translation [23,24]. In plant CMS systems, most cloned restorer genes are associated with PPR proteins [25,26,27,28,29]. In the present study, nine down-regulated and two up-regulated genes encoding PPR proteins were identified (Supplement 9), and these genes provide a significant molecular basis for subsequent studies of nucleo-cytoplasmic interactions.

### 3.4. DEGs Involved in MYB Transcription Factors

MYB (v-myb avian myeloblastosis vira 1 oncogene homolog) transcription factors play important roles in the regulation of gene expression during plant growth and mainly participate in primary and secondary metabolism, including anthocyanin [30] and flavonol biosynthesis [31]. Moreover, some MYB proteins are associated with anther development. *AtMYB103* is expressed specifically in anthers; decreased expression of *AtMYB103* causes early degeneration of the tapetum, and the majority of the resultant pollen grains are abnormal [32]. *AtMYB24* is induced by jasmonates (JAs) and is associated with filament elongation [33]. Overexpression of cotton *GhMYB24* in Arabidopsis causes flower malformation, shorter filaments, non-dehiscent anthers, and fewer viable pollen grains [34]. In the present study, 63 differentially expressed MYBs (Supplement 10) were identified. One of these, LOC107906095 (1.02-fold up-regulation), is orthologous to *GhMYB24.* These differentially expressed transcription factors might play a critical role in cotton pollen development. In addition, 405 other DEGs that encode transcription factors, comprising 184 up- and 221 down-regulated genes, were identified (Supplement 10). These differentially expressed transcription factors included “MYB-related”, “bHLH”, “AP2-EREBP”, and “C2C2-GATA”.

### 3.5. Other DEGs

Many other DEGs that might be associated with anther development were detected, and most of them were down-regulated, such as the anther-specific gene *LAT52* (LOC107961143, −4.03-fold down-regulation), which is expressed specifically in anthers and pollen [35]. Tetraketide α-pyrone reductase 1 (LOC108941000, −3.94-fold) and PHD (plant homeodomain) finger protein (LOC10784109, −6.15-fold) are related to pollen wall assembly. Calcium ions play important roles in the transmission of various signals, including those involved in the cell cycle, cell differentiation, and anther development [36]. Our data showed that 22 DEGs (Supplement 11) were associated with calcium ion regulation. Based on their function, these DEGs were divided into calcium-binding proteins, calcium-dependent protein kinases, calmodulin, calcium uniporter proteins, calcium-independent phospholipases, cation/calcium exchangers, calcium-responsive transcription coactivators, and calcium-transporting proteins.

## 4. Materials and Methods

### 4.1. Plant Materials

The plant materials used in this study were H276A (the CMS line) and H276B (the maintainer line). The plants were cultivated in the experimental field of Guangxi University under normal management conditions. Different-sized floral buds of the CMS line and its maintainer line were collected for microspore observations. The tetrad-stage (abortion stage, 4–5 mm in length) floral buds were frozen in liquid nitrogen and stored at −80 °C for RNA isolation.

### 4.2. Paraffin Sections and Microspore Development Observations

Different-sized floral buds were fixed in Carnoy’s fixation solution. After 24 h, the floral buds were washed with a graded ethanol series (95%, 85%, and 75%; 3 min per wash) and stored in 75% ethyl alcohol. The post-fixed floral buds were dehydrated with a graded ethanol series (75%, 80%, 85%, 90%, 95%, and 100%; 1 h per wash), hyalinized with a graded xylene series (100%, 60%, 30%, and 0%; xylene and paraffin; 5 h per wash) and then infiltrated in paraffin. After one month, the tissues embedded in paraffin were cut into 10-μm pieces. The paraffin sections were subjected to a series of 15-min washes with xylene (100%, 60%, 30%, and 0%; xylene and ethyl alcohol) and ethanol (100%, 95%, 90%, 85%, 80%, and 75%) and then dewaxed. Finally, the paraffin sections were stained with vanadium iron hematoxylin and observed using an electron microscope (Olympus BX53, Hataya, Japan).

### 4.3. RNA Extraction, cDNA Library Construction and Illumina Deep Sequencing

The total RNA from the floral buds (tetrad stage) of three H276A and three H276B plants was extracted using a Quick RNA Isolation Kit (Huayueyang, Beijing, China). The RNA concentration and integrity were checked using a NanoDrop 2000 spectrophotometer and an Agilent 2100 Bioanalyzer using the following threshold values: optical density (OD) 260/280 ≥ 1.8, OD260/230 ≥ 1.8, RIN ≥ 8, and 28S/18S ≥ 1.8. The same amount of total RNA (3 µg) was used for transcriptome cDNA library construction with a TruSeq™ RNA Sample Preparation Kit v2 (Illumina, San Diego, CA, USA). Briefly, RNA purification beads with oligo (dT) were used for the isolation of mRNA from the total RNA. A fragmentation buffer was added to break the mRNA into short templates. Double-stranded cDNA was synthesized using Super Script II reverse transcriptase (Invitrogen, Carlsbad, CA, USA) and purified with Agencourt AMPure XP-Medium (Agencourt, Carlsbad, CA USA). The short fragments were resolved with EB buffer for end reparation and A-tail addition. The short fragments were then connected with adapters. Following agarose gel electrophoresis, suitable fragments were selected as templates for PCR amplification. The quantification and qualification of the sample library were performed using the Agilent 2100 Bioanalyzer and an ABI StepOnePlus Real-Time PCR System, respectively. The cDNA library was then sequenced with an Illumina HiSeq 4000 platform. Solexa sequencing was performed at BGI Company (Shenzhen, China), and the raw sequencing files of these six samples (FASTQ files) are accessible from the NCBI Sequence Read Archive (SRA) database under Accession Number SRP114908.

### 4.4. Genome Mapping and Novel Transcript Prediction

Raw reads were defined as those that contain low-quality, adapter-polluted data and those that contain a high content of unknown base (N) reads; these noise reads should be removed prior to downstream analyses. After filtering, the remaining reads were mapped against the cotton reference genome (http://www.ncbi.nlm.nih.gov/genome/10704) using HISAT [37]. A transcript was considered novel if it contains features not present in the reference annotation. We used StringTie [38] to reconstruct the transcripts, and use cuffcompare to compare reconstructed transcripts to reference annotation, after that we select “u”, “i”, “o”, “j” class code types as novel transcripts [39]. We then used Coding Potential Calculator (CPC) [40] to predict the coding potential of the novel transcripts and merged the novel coding transcripts together with the reference transcripts to obtain a complete reference genome. All downstream analyses will be based on the complete reference genome.

### 4.5. Differential Expression Analysis

The expression level of each transcript was calculated with RSEM [41]. RSEM is a software package for estimating gene and isoform expression levels from RNA-Seq data. Differentially expressed genes (DEGs) were detected using the PoissonDis and DEseq2 methods. DEseq2 is based on a negative binomial distribution, and the DEseq2 analysis was performed as described by Love [42]. PoissonDis is based on the Poisson distribution, and the PoissonDis analysis was performed as described previously [43].

### 4.6. Functional Enrichment Analysis and Transcription Factor Prediction of DEGs

The NCBI blastall 2.2.23 program was used to query the final unigene set against the nr (non-redundant protein sequences, version 20160219) public protein database. Gene Ontology (GO, http://www.geneontology.org/ontology/obo_format_1_2/) classification and functional enrichment of DEGs were conducted using the hypergeometric distribution. Metabolic pathway analysis of the DEGs was performed with the Kyoto Encyclopedia of Genes and Genomes (KEGG) database (http://www.kegg.jp/) using the same method used for the GO analysis. Each open reading frame (ORF) of the DEGs was obtained with getorf [44]. All ORFs were then aligned to transcription factor domains (PlntfDB) using hmmsearch [45], and the transcription factors were identified according to PlntfDB methods (http://plntfdb.bio.uni-potsdam.de/).

### 4.7. Reverse-Transcription PCR (RT-PCR) and Quantitative Reverse-Transcription PCR (qRT-PCR)

One microgram of total RNA was reverse-transcribed into cDNA using a TransScript^®^ II One-Step gDNA Removal Kit and cDNA Synthesis SuperMix (Trans, Beijing, China). The relative expression levels of the DEGs were detected by qRT-PCR and estimated using the 2^−∆∆*C*t^ method. The *18s* gene of cotton served as the internal control. All primer pairs used in this study are listed in (Supplement 1). qRT-PCR was performed in a total volume of 15 μL that consisted of 7.5 μL of 2x TransStart Tip Green qPCR SuperMix (Trans), 4.9 μL of double-distilled water, 0.3 μL of each primer and 100 ng of cDNA. A C1000 Touch™ Thermal Cycler (Bio-Rad, Hercules, CA, USA) was used for the PCR, and the PCR conditions consisted of 95 °C for 30 s, 40 cycles of heating at 95 °C for 5 s and annealing at 60 °C for 30 s, and a temperature increase to 95 °C at a rate of 4.4 °C/s (each temperature gradient ran for 5 s). All qRT-PCR analyses consisted of three technical and three biological replicates.

## 5. Conclusions

In the present study, we comparatively analyzed the cytology and transcriptomes of the cotton CMS line H276A and its maintainer line H276B. The cytological results showed that microspore abortion occurred at the tetrad stage, the microspore disappeared at the late uninucleate stage, and the tapetum of the CMS line integrated during the process of microspore development. Therefore, during this development process, the microspore might not have obtained adequate nutrition from the tapetum, which might have led to CMS. The transcriptome results revealed 3603 DEGs between H276A and H276B, and these comprised 1363 up- and 2240 down-regulated DEGs in H276A compared with H276B. According to the bioinformatic analysis and previously reported data, key DEGs that might be associated with male sterility in H276A were identified, and these included DEGs involved in the TCA cycle, respiratory electron transfer chain, and oxidative phosphorylation, as well as DEGs that encode transcription factors and pollen wall assembly proteins. Our results provide a comprehensive foundation for understanding anther development and will accelerate the study of the molecular mechanism of CMS in cotton.

## Figures and Tables

**Table 1:** Transcriptome sequencing data quality and genome mapping.

Sample	H276A-1	H276A-2	H276A-3	H276B-1	H276B-2	H276B-3
Total Raw Reads (Mb)	50.42	53.42	51.82	51.81	51.82	51.82
Total Clean Reads (Mb)	40.38	44.48	44.73	44.58	44.25	44.37
Clean Reads Q20 (%)	98.06	98.01	98.39	98.38	98.39	98.34
Total Mapping Ratio (%)	73.34	74.20	75.20	76.04	75.60	76.18
Total Gene Number	57,228	57,777	57,428	56,938	56,929	56,629
Total Transcript Number	86,162	87,603	85,063	85,039	84,961	84,434
Novel Transcript Number	20,514	20,952	20,288	20,580	20,554	20,474

**Table 2:** Statistical enrichment analysis of Kyoto Encyclopedia of Genes and Genomes (KEGG) metabolic pathways (*p* ≤ 0.05, *Q* ≤ 0.05).

Pathway	Pathway ID	Genes with Pathway Annotation		*p*-Value	*Q*-Value
		DEGs (2683)	All Genes (56,363)		
Carbohydrate Metabolism					
Ascorbate and aldarate metabolism	ko00053	87 (3.24%)	747 (1.33%)	2.1883346 × 10^−14^	2.772849 × 10^−12^
Amino sugar and nucleotide sugar metabolism	Ko00520	132 (4.92%)	1789 (3.17%)	5.144044 × 10^−7^	1.306587 × 10^−5^
Galactose metabolism	Ko00052	93 (3.47%)	1264 (2.24%)	2.652517 × 10^−5^	3.142800 × 10^−4^
Pentose and glucuronate interconversions	Ko00040	119 (4.44%)	1711 (3.04%)	2.72211 × 10^−5^	3.142800 × 10^−4^
Fructose and mannose metabolism	Ko00051	48 (1.79%)	623 (1.11%)	0.0008306261	6.203606 × 10^−3^
Pentose phosphate pathway	Ko00030	44 (1.64%)	562 (1%)	0.0009706133	6.487784 × 10^−3^
Glycolysis/gluconeogenesis	Ko00010	62 (2.31%)	894 (1.59%)	0.00222321	1.344513 × 10^−2^
Global and Overview Map					
Metabolic pathways	ko01100	822 (30.64%)	14,040 (24.91%)	3.498244 × 10^−12^	2.221385 × 10^−10^
Biosynthesis of secondary metabolites	Ko01110	464 (17.29%)	8032 (14.25)	3.713275 × 10^−6^	5.239844 × 10^−5^
Biosynthesis of Other Secondary Metabolites					
Flavonoid biosynthesis	Ko00941	35 (1.3%)	266 (0.47%)	6.13195 × 10^−8^	2.595859 × 10^−6^
Stilbenoid, diarylheptanoid and gingerol biosynthesis	Ko00945	31 (1.16%)	240 (0.43%)	5.103317 × 10^−7^	1.306587 × 10^−5^
Flavone and flavonol biosynthesis	Ko00944	12 (0.45%)	105 (0.19%)	0.004277302	2.263406 × 10^−2^
Phenylpropanoid biosynthesis	Ko00940	91(3.39%)	1497 (2.66%)	0.01086005	4.755953 × 10^−2^
Metabolism of Terpenoids and Polyketides					
Zeatin biosynthesis	Ko00908	24 (0.89%)	162 (0.29%)	8.181436 × 10^−7^	1.731737 × 10^−5^
Limonene and pinene degradation	Ko00903	24 (0.89%)	210 (0.37%)	7.083635 × 10^−5^	7.496847 × 10^−4^
Carotenoid biosynthesis	Ko00906	21 (0.78%)	244 (0.43%)	0.006774023	3.308850 × 10^−2^
Transcription					
RNA polymerase	Ko03020	79 (2.94%)	962 (1.71%)	2.302535 × 10^−6^	3.878907 × 10^−5^
Energy Metabolism					
Nitrogen metabolism	Ko00910	22 (0.82%)	149 (0.26%)	2.443406 × 10^−6^	3.878907 × 10^−5^
Photosynthesis	Ko00195	22 (0.82%)	213 (0.38)	0.0005760129	4.572102 × 10^−3^
Lipid Metabolism					
Sphingolipid metabolism	Ko00600	33 (1.23%)	338 (0.6%)	8.44879 × 10^−5^	8.543074 × 10^−4^
α-Linolenic acid metabolism	Ko00592	27 (1.01%)	272 (0.48%)	0.0002803392	2.543077 × 10^−3^
Glycerolipid metabolism	Ko00561	65 (2.42%)	895 (1.59%)	0.0005644657	4.572102 × 10^−3^
Amino Acid Metabolism					
Tryptophan metabolism	Ko00380	26 (0.97%)	278 (0.49%)	0.0008792512	6.203606 × 10^−3^
Metabolism of Other Amino Acids					
Taurine and hypotaurine metabolism	Ko00430	6 (0.22%)	30 (0.05%)	0.002561534	1.478704 × 10^−2^
Glycan Biosynthesis and Metabolism					
Glycosaminoglycan degradation	Ko00531	23 (0.86%)	251 (0.45%)	0.002192842	1.344513 × 10^−2^
Other glycan degradation	Ko00551	42 (1.57%)	562 (1%)	0.002931596	1.618751 × 10^−2^
Glycosphingolipid biosynthesis (ganglio series)	Ko00604	16 (0.6%)	164 (0.29%)	0.005312663	2.698833 × 10^−2^
Glycosphingolipid biosynthesis (globo series)	Ko00603	8 (0.3%)	61 (0.11%)	0.00811891	3.818895 × 10^−2^
Transport and Catabolism					
Regulation of autophagy	Ko04140	34 (1.27%)	462 (0.82%)	0.008579786	3.891546 × 10^−2^

**Table 3:** qRT-PCR confirmation of the expression profiles of select genes.

Gene ID	Protein Identity	Fold Change	
		RNA-Seq	qRT-PCR
LOC107957220	Glycine-rich cell wall structural protein-like	4.92	5.02
LOC107903993	PPR protein At2g01860-like	2.81	0.70
LOC107893809	MADS-box CMB1	2.81	1.65
LOC107928908	Hyoscyamine 6-dioxygenase-like	2.73	2.71
LOC107940910	Ethylene responsive element binding protein 3	2.59	2.63
LOC107916224	Cell wall/vacuolar inhibitor of fructosidase 1-like	1.96	2.11
LOC107919764	Transcription factor MYB86	−3.21	−3.60
LOC107908692	Pentatricopeptide repeat-containing protein At3g11460	−3.38	−4.38
LOC107940452	Flavonol synthase/flavanone 3-hydroxylase-like	−3.86	−1.19
LOC107941000	Tetraketide α-pyrone reductase 1	−3.94	−3.50
LOC107961143	Anther-specific protein LAT52	−4.03	−2.74
LOC107945055	Chalcone synthase	−5.15	−5.72
LOC107900663	Bifunctional pinoresinol-lariciresinol reductase 2	−5.67	−3.43

Fold change refers to the expression in the CMS line compared with that in the maintainer line. A negative value indicates down-regulated expression in the CMS line.

## Supplementary Materials

Supplementary materials can be found at www.mdpi.com/1422-0067/18/11/2240/s1.

## Abbreviations

CMS	Cytoplasmic male sterility
DEGs	Differentially expressed genes
qRT-PCR	Quantitative reverse-transcribed PCR
PMC	Pollen mother cell
RNA-Seq	Transcriptome sequencing
TCA	Tricarboxylic acid cycle
KEGG	Kyoto Encyclopedia of Genes and Genomes
GO	Gene ontology
